# Targeting Cancer Stem Cells as the Key Driver of Carcinogenesis and Therapeutic Resistance

**DOI:** 10.3390/ijms24021786

**Published:** 2023-01-16

**Authors:** Refaat A. Eid, Muhammad Alaa Edeen, Eslam M. Shedid, Al Shaimaa S. Kamal, Mona M. Warda, Farag Mamdouh, Sohila A. Khedr, Mohamed A. Soltan, Hee Won Jeon, Mohamed Samir A. Zaki, Bonglee Kim

**Affiliations:** 1 Pathology Department, College of Medicine, King Khalid University, Abha P.O. Box 62529, Saudi Arabia; 2 Cell Biology, Histology & Genetics Division, Biology Department, Faculty of Science, Zagazig University, Zagazig 44519, Egypt; 3 Biotechnology Division, Zoology Department, Faculty of Science, Benha University, Al Qalyubia Governorate, Banha 13511, Egypt; 4 Biotechnology Department, Faculty of Agriculture, Benha University, Al Qalyubia Governorate, Banha 13511, Egypt; 5 Industrial Biotechnology Department, Faculty of Science, Tanta University, Tanta 31733, Egypt; 6 Department of Microbiology and Immunology, Faculty of Pharmacy, Sinai University, Ismailia 41611, Egypt; 7 Department of Pathology, College of Korean Medicine, Kyung Hee University, Seoul 02447, Republic of Korea; 8 Anatomy Department, College of Medicine, King Khalid University, Abha P.O. Box 62529, Saudi Arabia; 9 Department of Histology and Cell Biology, College of Medicine, Zagazig University, Zagazig 31527, Egypt

**Keywords:** cancer stem cells, carcinogenesis, resistance, therapeutic, cancer

## Abstract

The emerging concept of cancer stem cells (CSCs) as the key driver behind carcinogenesis, progression, and diversity has displaced the prior model of a tumor composed of cells with similar subsequently acquired mutations and an equivalent capacity for renewal, invasion, and metastasis. This significant change has shifted the research focus toward targeting CSCs to eradicate cancer. CSCs may be characterized using cell surface markers. They are defined by their capacity to self-renew and differentiate, resist conventional therapies, and generate new tumors following repeated transplantation in xenografted mice. CSCs’ functional capabilities are governed by various intracellular and extracellular variables such as pluripotency-related transcription factors, internal signaling pathways, and external stimuli. Numerous natural compounds and synthetic chemicals have been investigated for their ability to disrupt these regulatory components and inhibit stemness and terminal differentiation in CSCs, hence achieving clinical implications. However, no cancer treatment focuses on the biological consequences of these drugs on CSCs, and their functions have been established. This article provides a biomedical discussion of cancer at the time along with an overview of CSCs and their origin, features, characterization, isolation techniques, signaling pathways, and novel targeted therapeutic approaches. Additionally, we highlighted the factors endorsed as controlling or helping to promote stemness in CSCs. Our objective was to encourage future studies on these prospective treatments to develop a framework for their application as single or combined therapeutics to eradicate various forms of cancer.

## 1. Introduction

According to the GLOBOCAN research study, the leading causes of cancer-related deaths in 2018 were lung (1.76 million deaths), stomach (783,000 deaths), liver (782,000 deaths), breast (627,000 deaths), and colorectal cancers (551,000 deaths), as well as blood malignancies such as leukemia (309,000 deaths) [1]. These kinds of malignancies are heterogeneous tumors composed of cells with a wide range of stem cell characteristics. Santosa et al. [2] recognized this cell subpopulation in 1877 and remarked on its embryonic nature. Cancer stem cells (CSCs) or tumor-initiating cells (TICs) are now thought to be crucial in tumor initiation, progression, and therapeutic resistance [2]. 

The constitution of tumor cell subpopulations is altered following treatment with chemotherapy or radiation. Tumor cells with high proliferative potential are initially targeted and eliminated, thereby leading to a reduction in the tumor size while CSCs persist [3]. Therapy-induced senescence (TIS) of several cancer cells may even alter the tumor microenvironment (TME) in a tumor-promoting manner via the senescence-associated secretory phenotype (SASP). CSCs are challenging to eradicate and can lead to cancer recurrences [4]. Nonetheless, CSCs can be regenerated in a therapeutically pressurized and altered microenvironment. To be more precise, these cells do not come from CSCs but rather from senescent cancer cells induced by treatment interventions [5,6,7]. Consequently, it is crucial to identify these cells and discover their origin throughout tumor development and recurrence.

This article highlights the importance of an extensive examination of malignancies, particularly in the post-therapeutic stage, which is not yet routinely performed in clinics. Establishing the significance of biomarkers that examine many characteristics, including CSCs’ phenotypes, senescence, and TME composition, would enable the diagnosis of therapy-resistant CSCs that induce cancer relapses. This review discusses the exact and timely eradication of challenging cells by employing targeted cellular therapeutic strategies as second-line therapy.

## 2. Cancer Stem Cells (CSCs)

CSCs are a small subpopulation of cells inside a tumor that can self-renew and develop into all cell lineages in the heterogeneous TME [6]. Despite being a well-established theory, the concept that tumor progression is initiated by a small number of “stem-like” cells has recently received much attention. In 1855, a German doctor named Rudolf Virchow suggested the theory and argued that it contributed to cancer development [7].

It has been hypothesized that tumors derived from CSCs follow a unidirectional hierarchy in which only the CSC subpopulation can initiate tumor progression [8]. It also has been hypothesized that at the moment of tumor development, CSCs divide asymmetrically to preserve the CSC pool [9]. These asymmetric divisions yield transiently amplifying cells that undergo symmetric divisions; hence, such cells have a high potential for proliferation [8,9]. The AML datasets, the hierarchical model presented by Bonnet and Dick [10], are most likely an oversimplified explanation. It is currently assumed that the organization of CSCs (in both solid and hematological malignancies) is more complicated [11].

**Figure 1:**
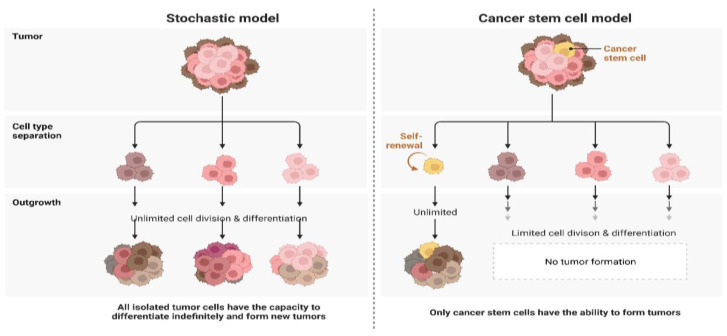
Schematic representation of the stochastic and CSC models of carcinogenesis.

Contrary to the CSC model, which proposes that only a small subpopulation of cells can enhance cancer development and growth, the clonal evolution model proposes that genetically unstable cells accumulate genomic as well as genetic variations over time, thereby leading to a rise in tumor aggressiveness, therapeutic resistance, and heterogeneity [12]. These models are not mutually exclusive, which may be justified by the theory of cellular plasticity, which proposes that distinct cellular states may interconvert (Figure 1) [13].

## 3. Origin of CSCs

### 3.1. Cell Fusion

Cell fusion is a typical physiological process that occurs in several organisms and performs crucial functions in fertilization as well as organ system development. Nevertheless, cell fusion may result in aneuploidy and malignancy when it gets out of control [14].

The latest stem cell biology results concerning tissue regeneration and somatic cell transdifferentiation have revolutionized the cell fusion concept of carcinogenesis [15]. Cell fusion generates hybrids with twice the number of chromosomes and centrosomes, a situation that can result in aberrant chromosomal segregation and aneuploidy. Unlike the traditional paradigm of tumorigenesis based on the linear accumulation of mutant alleles, cell fusion effectively generates non-linear patterns of genetic rearrangements and related phenotypic changes [14,16].

Bjerkvig et al. and Dittmar and collaborators [16,17] postulated that cell fusion is connected to the development of CSCs. 

Multiple findings further reinforced the validity of this hypothesis. Chronic inflammation, a significant risk factor for carcinogenesis, significantly boosted the fusion of BMDCs with differentiated adult tissue cells [17,18]. In an animal model of gastric cancer caused by persistent inflammation from Helicobacter pylori infection, it was discovered that BMDCs replaced the malfunctioning epithelial lining of the gastric mucosa and ultimately led to the development of gastric cancer [19]. However, no clear evidence of cell fusion was discovered in this research study; supplementary experimental studies that utilize fusion-tracing approaches, including Cre-mediated activation of a LacZ reporter preceded by a lox-franked stop signal, must therefore be carried out to examine the possible role of cell fusion in the creation of CSCs and the initial development of tumors.

### 3.2. Horizontal Gene Transfer

Horizontal gene transfer (HGT) was first discovered in microbes in the late 1940s, and nearly 20 years later, it was hypothesized to have a role in the adaption of multicellular eukaryotes. Since then, technology for determining HGT has evolved to expose the incredible amount and significance of HGT in the diversity of viral, prokaryotic, and eukaryotic gene encoding. HGT, rather than autochthonous gene duplication, has now been confirmed as the origin of many apparent gene duplications [11].

HGT is prevalent in bacteria and fungi, enabling organisms to develop various adaptations, including antibiotic resistance. HGT involves transferring donor DNA to recipient cells, inserting acquired sequences into the recipient’s genome, and ultimately expressing the inserted genes in a beneficial manner [12]. Transformation, transduction, and conjugation are mechanisms that can be used to complete the first two phases. HGT occurs in eukaryotic cells when DNA is passed from dead cells to recipient cells via phagocytosis or endocytosis [13].

Mutations in somatic cells can trigger apoptosis and DNA fragmentation. The latter can be phagocytosed or endocytosed by other somatic cells, resulting in nuclear reprogramming and the generation of new aggressive cells. Different tumor cells may be able to take up fragmented DNA [14].

Epstein–Barr virus (EBV) might be transmitted from integrated cells into the nucleus of phagocytosing cells, where EBV-encoded genes are produced both at the mRNA as well as protein levels [15]. Apoptotic bodies from cancer cells can lead to the formation of colonies in vitro and tumors in vivo by p53-deficient fibroblasts [14]. It is now thought that whole or pieces of chromosomes could be transmitted to tumor cells via phagocytosis. The fact that tumor cells have such a high phagocytic capacity suggests that genetic material transfer may be significant in CSCs’ creation, tumor initiation, and development [16].

### 3.3. Dedifferentiation in Cancer Cells

Another aspect that promotes the creation of CSCs is cellular dedifferentiation. A differentiated cancer cell can dedifferentiate into a CSC in response to various circumstances, such as trauma, stress, and hypoxia, thereby resulting in the initiation and progression of cancer [17]. According to a recent study, glioma cells may dedifferentiate into glioma stem-like cells (GSCs) in response to stress and hypoxia-induced HIF1 signaling. Additionally, it has been proven that angiocrine chemicals such as nitric oxide (NO) induce glioma cells to dedifferentiate and form GSCs [18]. Additionally, ionizing radiation endowed cancer cells with stem-like properties, facilitating metastatic dissemination. EMT has been shown to cause dedifferentiation of pancreatic ductal cells and their acquisition of CSC characteristics. These data suggest that dedifferentiation is critical in developing CSCs in various types of malignancies [17].

### 3.4. Tumor Microenvironment (TME)

**Figure 2:**
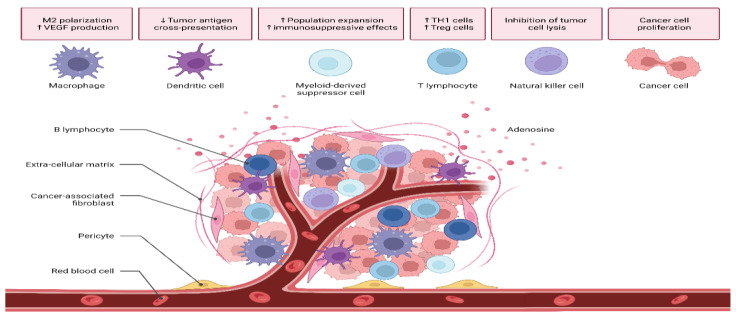
A diagrammatic representation of the microenvironment of a tumor highlighting the multiple cell phenotypes.

The TME performs a critical function in the development and progression of tumors. TME constituents can be classified as either cellular or acellular [19]. Tumor stromal cells, CAFs, TECs, pericytes, B lymphocytes, T lymphocytes, TAMs, TAAs, and CSCs make up the cellular components of the tumor. In addition to the ECM and soluble compounds, the acellular element includes exosomes and other extracellular vesicles (Figure 2) [20].

**Figure 3:**
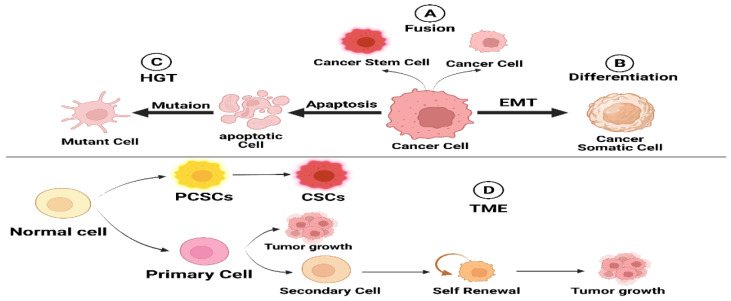
Schematic diagram summarizing the potential origins of CSCs.

Enhanced cancer growth and metastatic spread result from bidirectional interaction between cells in the TME. Cancer cells need stromal cells to generate their TME. The TME is established whenever the underlying tissue structure is alternated by growth-promoting signals and intermediate metabolites released by the cellular component. ECM operates as a physical scaffolding for all cells, allowing them to reside in the TME and migrate in and out dynamically, thereby enabling tumors to proliferate and metastasize. An ECM is a structure composed of fibrillar proteins, auxiliary proteins, and chemicals that give structural and biochemical support to cells. Fibrillar collagen is the central element of the ECM, and its structure and mechanical properties have an important influence on the cellular phenotype (Figure 3) [21,22].

## 4. Features of CSCs

### 4.1. Autophagy

Autophagy is a tightly controlled, conserved catabolic process that serves as a cell-survival mechanism in response to cellular stressors such as malnutrition, hypoxia, and chemotherapy/radiotherapy [20]. Abnormalities in the autophagy mechanism have been linked to neurodegeneration, muscular dystrophy, and possibly several malignancies [23].

It is thought that CSC populations must rely on their nearby surrounding microenvironment to survive. In recent years, autophagy has emerged as a crucial mechanism for the maintenance and resistance of CSCs [24]. Autophagy has also been implicated in maintaining the CSC/normal stem cell balance as stable [25].

Curcumin promoted the maintenance of colon CSCs, as demonstrated by Kantara et al. [26]. Curcumin significantly suppressed the expression of stem cell markers when used at therapeutic doses. Curcumin, which is commonly used in curry, surprisingly boosted CSC multiplication and autophagic survival. Curcumin destroyed in vitro spheroid cultures; however, cisplatin promoted their rapid regeneration within 30–40 days. These findings suggest that autophagy provides a survival advantage that allows colorectal cancer to persist for extended periods [26].

Furthermore, Sanchez et al. [27]. revealed that mesenchymal stem cells devoid of serum (SD-MSCs) encouraged MCF-7 tumor development. Tumors injected with SD-MSCs displayed increased cellularity, reduced apoptotic cell death, and increased differentiation. Beclin1 labeling revealed autophagic regions bordered by growing cells. In vitro experiments showed that SD-MSCs survived through autophagy and released paracrine mediators that assist cancer cells after nutrient/serum deficiency [27].

To sum up, the biological mechanisms behind autophagy are still unclear. While the outcome of autophagy remains unknown, it may be influenced by variables such as stimulation, cell type, and microenvironment. Consequently, identifying the physiological significance of autophagy in CSCs and developing therapeutic methods would necessitate a comprehensive knowledge of its underlying molecular mechanisms, signaling cascades, and the participation of regulatory circuits. Interesting novel autophagy modulators for safer, highly effective cancer treatments deserve continued research.

### 4.2. Self-Renewal, Differentiation, and Tumor Recurrence

Self-renewal and differentiation are two major characteristics of CSCs. CSCs have been demonstrated to preserve their subpopulations during their lifetimes via a self-renewal mechanism and differentiate into phenotypically distinct neoplastic cells that contribute to the tumor mass [28]. Self-renewal is a cell division in which one or both descendent cells continue to function as stem cells that maintain the population [29]. Self-renewal and differentiation of CSCs are tightly regulated processes that are influenced by both intrinsic and extrinsic signals. Extrinsic variables, including the interactions between CSCs and stromal cells, influence CSC self-renewal [30]. Gene mutations are a great example of the intrinsic variables that can trigger the uncontrollable stimulation of stem cells’ self-renewal molecular pathways, such as Wnt, Notch, and Hedgehog; this results in the transformation of normal stem cells into CSCs [31].

Numerous studies indicated that the Wnt/β-catenin signaling pathway was required for stem cell self-renewal. It has been demonstrated that inhibiting the Wnt pathway with pyrvinium pamoate decreases CSC self-renewal and spread [32]. Let-7c is a miRNA that functions as a tumor suppressor by reducing CSC self-renewal in estrogen receptor (ER) + ve breast cancer by downregulating ER expression and inhibiting Wnt signaling. Let-7 was also demonstrated to increase tamoxifen’s anticancer efficacy via controlling CSC self-renewal [33]. Furthermore, a recently published research paper revealed that JAK/STAT3-pathway-regulated fatty acid oxidation is necessary for BCSC self-renewal. Fascin, an actin-binding protein, is essential for CSC self-renewal via the activation of Notch signaling [34].

### 4.3. Induction of Angiogenesis 

The formation of a vascular network is essential for cancer progression and metastasis [35,36]. If there are inadequate blood arteries to provide appropriate nourishment to tumor cells, the tumor volume cannot reach 2–3 mm^3^, and organ structures and functions are similarly impaired [37,38]. Since endothelial cells are essential for creating vasculature, with a lack of endothelial cell proliferation [39], there will be no clinically apparent tumors or secondary blood vessels. Consequently, antitumor angiogenesis treatments are a prominent focus of cancer therapy research [40].

Vasculogenesis and angiogenesis are the primary mechanisms that create new blood vessels [41,42]. Vasculogenesis is described as transforming endothelial precursor cells (EPCs) or angioblasts into endothelial cells and developing new primitive vascularization. At the same time, angiogenesis is the process by which new capillaries develop from already-present blood vessels, and it can occur either through sprouting or intussusception [43]. Wang et al. [44] revealed that CSC-derived endothelial cells might contribute to tumor angiogenesis. Stemness marker ALDH1A1 increased tumor angiogenesis in MCF-7 breast cancer cells via retinoic acid/HIF-1/VEGF signaling. ALDH1A1 expression in breast cancers generates a favorable microenvironment by increasing angiogenesis through a retinoic-acid-dependent mechanism. Ciccone et al. [45] revealed that ALDH1A1 increased tumor angiogenesis through retinoic acid/HIF-1/VEGF signaling in MCF-7 breast cancer cells. Maiti et al. [46] employed a Class I histone deacetylase inhibitor to reduce vasculogenic mimicry in triple-negative breast cancer (TNBC) cells by enhancing the expression of tumor suppressor and antiangiogenesis genes.

### 4.4. CSCs Promote Metastasis

Metastasis is the migration of cancer cells from the primary tumor to other organs [47]. Multiple signaling pathways govern the metastatic process with great precision in a complicated system where tumor cells access the circulation by invading and spreading to remote organs to form new tumors [48]. As per the accumulating data, CSCs probably are crucial for metastasis due to their innate anoikis resistance [49]. Anoikis is a form of controlled cell death that occurs when anchorage-dependent cells separate from their substrate [50]. Many cancer cells die in the bloodstream, while CSCs survive and establish distant metastatic tumors [49,51]. It has been demonstrated that the CXCL12-CXCR4 signaling pathway is involved in the development of breast cancer. This pathway is responsible for promoting tumorigenesis and angiogenesis, initiating cancer cell invasion in vitro, and directing malignant cells to the areas where they will metastasize [52]. Bernat-Ablett et al. [53] demonstrated that the CSCs of mice with advanced squamous cell carcinoma expressed the SDF-1 receptors CXCR4 and CXCR7. Signaling via PDGFR leads to the increased production and release of SDF-1 in L-CSCs as well as autocrine activation of the pathway in concern. Autocrine SDF-1/CXCR4 signaling promotes in vivo lung metastasis by stimulating lung CSCs’ proliferation and survival in addition to acting as a mediator of PDGFR-induced invasion. Activating CXCR4 signaling by SDF-1 causes mammosphere development and resistance to anoikis in breast cancer cells [54]. Wnt activation has also been demonstrated to be greater in cells resistant to anoikis [55].

### 4.5. Radiation and Chemoresistance

Radiotherapy, also known as RT, is the treatment of preference for the vast majority of solid tumors, such as glioblastomas and lung and breast cancers. The first obstacle that needs to be overcome in RT is the radioresistance of tumor cells, which is implicated in the development of both locoregional relapse and distant metastasis [56]. High-energy radiation kills cancer cells by producing extensive DNA damage or creating free radicals [57]. CSCs are resistant to a wide variety of treatments, which include radiotherapy; this is attributed to their features, particularly their capacity to repair DNA damage, produce few reactive oxygen species (ROS), and divide only slowly; these features are regulated by altering/increasing DNA repair enzymes, checkpoint proteins, and free radical scavenging [58,59]. ROS causes DNA damage by breaking strands and oxidizing bases; this damage, if not repaired, can lead to apoptosis or oncosis [60,61]. Radiotherapy activates NFB, a transcription factor that mediates radioresistance in pathological expression; this shows that radiotherapy-induced Her^2+^ CSCs may cause therapeutic resistance and severe relapse [62].

Keap1-Nrf2 regulates ALDH and contributes to radioresistance in CSCs [63]. STAT3-dependent, radiation-induced cellular plasticity alterations might reduce radioresistance in TNBC and improve treatment outcomes [64]. Therefore, therapy with regularly administered medications frequently increases the percentage of CSCs. Additionally, it has been demonstrated that the establishment of drug resistance is correlated with a more significant portion of the CSC population [65].

It is well-recognized that CSCs employ a variety of mechanisms to defend themselves against chemotherapeutic agents and ionizing treatments [66]. Despite extensive previous research, the processes by which cancers develop chemoresistance remain unknown. Tumor diversity directly results from CSCs and is a fundamental hallmark of therapeutic resistance [65].

**Figure 4:**
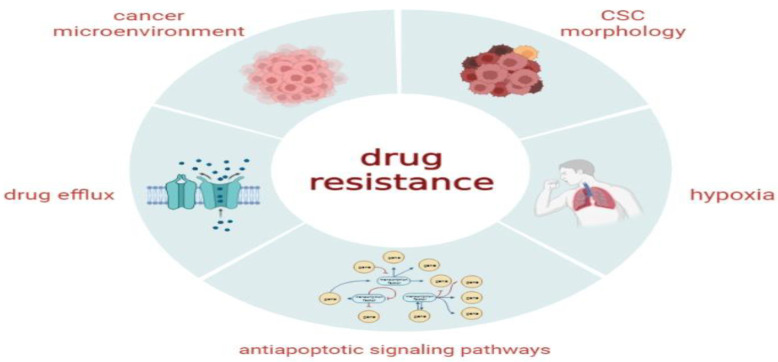
Potential molecular mechanisms that contribute to intrinsic or acquired treatment resistance to conventional therapeutic approaches for various forms of cancer.

ALDH1 is a member of the NADP+-dependent enzyme superfamily characterized by its physiological and detoxifying functions in CSC self-defense. The ALDH1 enzyme converts aldehyde to carboxylic acids, which aggregate due to chemotherapy, radiation, or other factors (Figure 4).

## 5. Isolation Techniques of CSCs

To examine the fundamental properties of CSCs, scientists must first extract, purify, and characterize these populations through technologies that separate them from the rest of the malignant cell population. This objective is exceedingly difficult to accomplish because CSCs comprise a small proportion of the total cell population in a tumor and might express the same cell surface markers as their fully differentiated counterparts [67]. The side population assay (SP), specific expression of cell surface markers, tumorigenicity, ALDH, tumorsphere, stem gene expression, transcription factors, and label-retention assays such as PKH staining are among the most effective techniques for CSC isolation [68]. These potential CSCs could be biologically validated in vivo using a sequential transplantation procedure to measure tumorigenicity and self-renewal capacity [69].

### 5.1. Isolation with Surface Markers

CSCs are characterized by a few protein effluxes. Various cellular surface biomarkers are crucial for detecting and targeting CSCs in diverse pathologies. The articulation patterns and level of articulations of these biomarkers vary amongst tumor masses; still, no clear signals have been shown. In cancer research, markers of the CSC subpopulation such as proteins are typically employed to create a profile for differentiating the CSC population from the mixed population of cells [70]. CSCs can be tagged, sorted, and tested/manipulated via FACS or magnetic cell sorting (MACS) [52,71]. CD34 + CD38− acute myeloid leukemia (AML) cells were used to identify CSCs [72]. From then on, CSCs have been found in solid tumors, especially breast cancer [73]. CD24, CD44, CD133, EpCAM, CD49f, CD90, and CD61 are commonly employed to identify and distinguish CSCs either alone or in combinations [68]. When CD24 − CD44+ BCSCs were administered into the NOD/SCID mice mammary fat pad, they exhibited their fundamental features involving self-renewal and differentiation, thereby resulting in the development and progression of engrafted breast tumors [74]. While the CD24 − CD44+ phenotypic markers have been extensively used to identify CSCs in various types of tumors, most notably basal-like tumors [75], a previous study reported that both CD24 − CD44+ and CD24 + CD44 + cell populations in ER-negative breast tumors were oncogenic [76].

CSCs detected by cellular markers exhibit a greater specificity than those separated using functional testing [77]. This strategy, nevertheless, does have certain limitations. First, most surface markers used to detect CSCs were primarily employed to identify other types of stem cells, including embryonic and adult stem cells, which suggested concerns regarding their specificity and reliability [78]. Furthermore, the lengthy and complicated approach to CSC separation using surface markers might degrade the surface markers throughout the specimen preparation, resulting in a decreased number of isolated CSCs [79]. Ultimately, there is no uniform marker for distinguishing CSCs; the expression of markers depends on several factors, particularly the culturing conditions and the microenvironment characteristics [80].

### 5.2. Side Population Assay (SP)

Stem cells are distinguished by the upregulation of members of the ATP-binding cassette (ABC) transporter protein family, which can use ATP to pump a variety of chemicals, particularly medications, out of the cells [68]. Moreover, these pumps remove potentially hazardous chemicals, which contribute to the detoxification process of the cell [81]. 

These efflux pumps also include chemotherapeutic drugs as substrates, proving that CSCs have developed a mechanism for drug resistance [81]. The overexpression of these ABC transporter proteins in CSCs not only identifies them as an SP but is also considered a necessary factor in CSC-mediated drug resistance [82]. SP cells were also discovered in distinct subtypes of human breast cancer cells with a greater propensity for carcinogenesis than cells that did not efflux essential dyes adequately [83].

Stem cells are routinely separated through FACS techniques and the Hoechst SP procedure [84]. Hoechst is a fluorescent dye that binds to all nucleic acids but prefers the AT-rich areas of the minor groove of DNA. Hoechst 33,342 can pass through the plasma membranes of live cells [85]. Hoechst is triggered at 405 nm, and the blue signal emitted is captured using a 450/40 nm bandpass filter. Using a 610/20 nm filter, the red fluorescence is simultaneously obtained. Hoechst dye can be characterized as a population of negative cells for Hoechst blue and red due to the SP’s propensity for extrusion [85].

### 5.3. Label-Retaining Methods (Lipophilic Dyes)

The cell membrane label-retaining assay was recently developed as a unique in vitro characterization approach for CSCs [86]. A PKH fluorescent dye series is used in this test because it has a fluorophore-conjugated peptide backbone that is irreversibly bound to the phospholipid in the cell membrane [87]. When a cell divides, these colors are evenly distributed across the daughter cells. Cells that divide slowly retain the dye, but cells that divide rapidly lose or dilute the dye from the membrane. The asymmetric division of CSCs was demonstrated by the use of the PKH26 labeling approach [88]. Unlike rapidly dividing differentiated daughter cells, CSCs spend longer in dormancy and asymmetric division before losing their label [12]. Therefore, a PKH dye label-retaining mammosphere test was applied to detect CSCs.

### 5.4. Tumorigenicity

CSCs are especially remarkable due to their ability to grow serially transplantable tumors in immunocompromised hosts, mimicking the primary tumor and generating cells of multiple lineages that comprise the tissue of origin. This is a prerequisite for establishing the identity of CSCs; hence, tumorigenicity is recognized as the benchmark test for evaluating the biology of CSCs and their therapeutic responsiveness [89,90]

Limited dilution assay (LDA) is the optimal tumorigenicity technique for evaluating the percentage of active CSCs [91]. With the invaluable extreme limiting dilution analysis (ELDA) software, it is possible to detect the proportions of subpopulations with 0–100% responsiveness [92].

The number of cells, the site of implantation, and the incubation duration all affect the outcome of this procedure. This method is not appropriate for high-throughput screening [92].

### 5.5. Aldehyde Dehydrogenase Assay

ALDH isoenzymes mediate the intracellular aldehyde oxidation process in the cytosol. This process is thought to be necessary for stem cell differentiation and consequent organogenesis as well as homeostasis because ALDH1 isoforms regulate the conversion of retinol to retinoic acid in both normal and CSCs for detecting CSCs and tumor-infiltrating cells (TICs); therefore the fluorescent ALDEFLUOR test was established [93].

A poorer overall survival rate was shown to be associated with higher levels of ALDH1A1 activity in patients with colorectal cancer even though the expression of ALDH1A1 and A3 in CSCs is assumed to be necessary for ALDH activities [94]. Geinster et al. [94].were the first to employ the ALDEFLUOR assay on normal and cancer tissues, while other groups applied it to cancer cell lines. Fluorophores that can cross the plasma membrane of intact and living cells, such as BAAA, can identify cells with ALDH1 activity [95,96]. Intercellular ALDH can convert BAAA to the fluorescently labeled BodipyTM-aminoacetate (BAA−), which is retained in cells when inhibitors (verapamil) are added to the assay system and impede the exclusion of BAAA via ABC transporter proteins [97].

This technique may commonly identify and separate viable cells because BAA− may be retained in only viable cells with intact cellular membranes [98]. The ALDH inhibitor diethylaminobenzaldehyde is required as a negative control for all assays in which ALDEFLUOR-stained cells are used [99]. Cell subpopulations with elevated ALDH1 expression can be identified by Aldefluor labeling or FACS analysis [100]. The Aldefluor test and FACS analysis indicated that ALDH1-positive cells had a more substantial capacity for sphere formation, self-renewal, tumorigenicity, and expression of stemness genes than ALDH1-negative cells [96]. Aldefluor positivity has been found to label and detect CSCs in their associated tumor tissue slides when paired with additional unique stem cell surface markers such as CD133+ and CD24 − CD44+ [101].

### 5.6. Spheroid Formation Assay

When cultured in non-adherent serum-free conditions, CSCs can generate multicellular three-dimensional (3D) spheres. These spherical morphologies have a well-rounded shape, a minimal size, the ability to survive as free-floating cultures, and the presence of cancerous cells [102]. It has been determined that the sphere-formation assay is the gold standard for identifying CSCs and assessing their pluripotency [103]. During the culture process, previously detached cells from the central nervous system (CNS) form spherical colonies and produce neurons and astrocytes. The latest research has shown that the population of CSCs and TICs may be significantly enhanced if certain mitogens that enhance CSC proliferation in non-adherent circumstances are present. These mitogens include epithelial EGF (epidermal fibroblast growth factor) and basic fibroblast growth factor (bFGF) [104]. In this culture, immature or undifferentiated cells develop gradually over time and eventually clump together into non-adherent clusters known as tumorspheres. Non-malignant cells, often differentiated, die out in opposition [102].

### 5.7. Stemness Gene Expression and Transcriptional Factors

CSCs are also mainly detected via the expression of stemness genes [103]. The transcription factors OCT4, Sox2, and Nanog are crucial to maintaining pluripotent embryonic stem cells and germ cells. These genes are commonly expressed in committed progenitors as well. Their emergence is probably a consequence of carcinogenic transformations and is not confined to the CSC population. Unfortunately, there are multiple such genes. The precise number essential to impart stem cell characteristics and the level of expression needed is uncertain. Gliomas and breast cancer have primarily been associated with a higher expression of these genes [105]. Bmi-1, Snail, and Twist are three other transcriptional factors that should be considered.

Snail and Twist are essential in promoting EMT. Snail induces EMT by downregulating E-cadherin, cytokeratin, and desmoplakin expression while upregulating vimentin and fibronectin. Twist, like Snail, promotes EMT, thereby allowing cancer invasion and metastasis. Twist is upregulated in breast cancer [106]. Additionally, overexpression of two transcription factors results in EMT developing stemness properties, including enhanced expression of stemness surface markers, enhanced capacity to establish spheres and create tumors in xenografts, and enhanced invasiveness and metastatic potential. As a result, Snail and Twist play critical roles in CSC survival [107].

## 6. Signaling Pathways Governing CSCs’ Behavior

**Figure 5:**
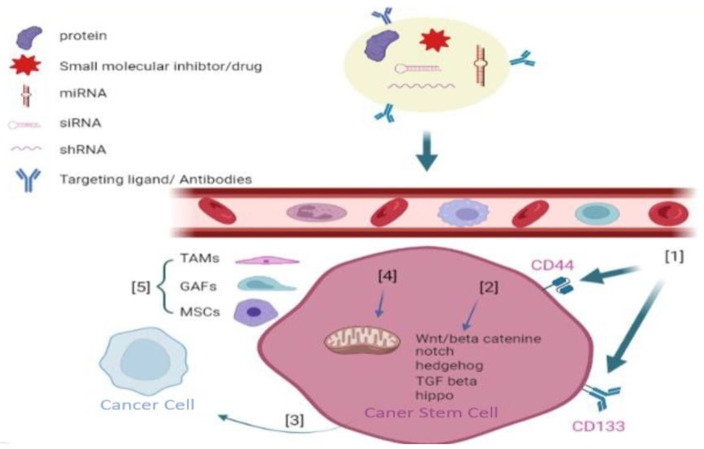
CSCs proliferate quickly via SRPs such as Nanog, Notch, Hedgehog, Wnt, and JAK/STAT dysregulation.

In carcinogenesis or CSCs, a significant number of signaling pathways that implement the survival, proliferation, self-renewal, and differentiation capabilities of normal stem cells are either abnormally stimulated or inhibited. These intricate pathways are regulated by a large number of genes—both endogenous and external—as well as microRNAs. These signaling pathways could also drive downstream gene expression in CSCs, including the production of cytokines, growth factors, genes involved in programmed cell death and antiapoptosis, and genes involved in proliferation and metastasis. These signaling pathways are not a single regulator but rather intricate networks of signaling mediators that act together to control the formation of CSCs [108,109]. Consequently, this section aims to explain how signaling pathways regulate the expansion of CSCs (Figure 5).

### 6.1. Wnt Signaling Pathway in CSCs

Wnts are large protein ligands that govern a range of biological processes, particularly the establishment of cell polarity and cell fate; with 19 Wnt ligands and over 15 receptors, the Wnt pathway is extraordinarily intricate and evolutionarily stable [110]. Wnt signaling includes canonical (via the FZD-LRP5/6 receptor complex, resulting in β-catenin dysregulation) and non-canonical (via FZD receptors and/or RYK/ROR1/ROR2 coreceptors, triggering PCP, RTK, or Ca^2+^ signaling cascades) [111]. In canonical Wnt signaling, glycogen synthase kinase 3 phosphorylase-catenin is inhibited in the absence of wnt ligands (inactive Wnt signaling state), thereby leading to β-catenin degradation via β-TrCP200 ubiquitination as well as β-catenin translocation from the cytoplasm to the nucleus [112]. With the existence of Wnt ligands (e.g., Wnt3a and Wnt1), fzd receptors and LRP coreceptors are joined with the ligands. Both GSK3 and CK1 phosphorylate the LRP receptor. The Axin complex facilitates the entry of β-catenin into the nucleus. When paired with LEF/TCF, β-catenin enhances the induction of histone-modifying coactivators known as Pygo, CBP/p300, BCL9, CBP/p300, and BRG1. β-catenin is not required for non-canonical Wnt signaling to occur [112].

Dvl is triggered by Wnt ligands binding to the ROR-Frizzled receptor via Wnt/PCP signaling. The binding between the small GTPase Rho and the cytoplasmic protein DAAM1 are both inhibited by DvlRac1 and Rho, which are two small GTPases that work together to activate JNK (c-Jun N- terminal kinase) and ROCK (Rho-kinase); this occurs in cytoskeletal reorganization and transcriptional responses [111]. G-protein-stimulated phospholipase C activity stimulates Wnt/Ca^2+^ signaling, resulting in intracellular calcium flux, transcriptional responses, and calcium-dependent cytoskeletal [113]. Wnt stimulation tends to cause dormant CSCs to become active CSCs, encouraging cell cycle progression by raising the synthesis of downstream cyclin D1, MYC, and β-catenin [114]. Wnt signaling is also implicated in the control of CSC apoptosis. Dickkopf-related protein 2 reduces β-catenin activity in CSCs, triggering cells’ G0/G1 arrest and death [115].

### 6.2. Hedgehog (Hh) Signaling

Hedgehog signaling is necessary to regulate stem and progenitor cell proliferation, fate, and regenerative capacity [116]. The Hh signaling pathway consists of the transmembrane protein receptor PTCH, the transmembrane protein SMO, transduction intermediary molecules, and the downstream molecule GLI [117]. The elements of the Hh signaling pathway each have a unique purpose. Positive regulators include SMO (a membrane protein) and PTCH (a transmembrane protein). PTCH is classified into two forms: PTCH1 and PTCH2,38, which share 73% homology. In invertebrates, the effector protein GLI is subdivided into three subtypes: Gli1, Gli2, and Gli3, each of which performs a distinct role [114].

Once Hh attaches to PTCH, it changes its spatial orientation, thereby allowing SMO to recruit GLI to the cell nucleus, where it promotes cellular growth, proliferation, and differentiation [113].

As per previous studies, human cancers exhibit an abnormal activation of the Hh signaling system [118]. Hh signaling serves a variety of activities in many types of cancer; it plays a critical role in the development of tumors by inducing tumorigenesis, promoting tumor progression, and controlling the remaining cancerous cells post-treatment [119].

In CSCs, Hh signaling components are highly expressed [118]. When expressed in CSCs and medulloblastomas, WIP1, a nuclear Ser/Thr phosphatase, increases the transcriptional activity, protein stability, and nuclear localization of Gli1 [114]. In basal-like breast cancer (BLBC), upregulation of FOXC1 improved CSC characteristics via Gli2 [120]. The self-renewal of mammary epithelial stem cells is governed by p63 when their levels are higher than those of normal progenitor cells [121].

Even lncRNAs associated with Hh signaling (LncRNA-Hh) contribute to the enhancement of OCT4 and SOX2 expression, which is required for CSC survival. The opposing impact of silencing lncRNA was observed, which suggested that it plays a vital role in regulating stemness preservation [122].

### 6.3. Notch Signaling

NOTCH pathway mediates interactions between two neighboring cells; the first cell has a ligand, and the second contains a programmable receptor capable of combining with the ligand. In human bodies, four heterodimeric transmembrane Notch receptors have been discovered that respond to the transmembrane ligands Jagged (JAG) 1 AND 2 and Delta-like (Dll) [123,124].

Notch receptor is stimulated when it binds to a ligand provided by an adjacent cell, which activates Notch signaling as the ligand approaches the receptor. Attaching to a ligand in an adjacent cell triggers the Notch, and the ligand moves toward the receptor, thereby activating Notch signaling. The signal is then rapidly transmitted from the cell membrane to the nucleus and nearby cells, which initiates a series of biochemical events that may affect the cell [125].

Different cancers express distinct Notch ligands and receptors. Furthermore, Notch serves two functions. The first is an oncogene, and the second is a suppressor gene. To begin, Notch is overexpressed as an oncogene in various cancers, including breast and pancreatic [124]. At the same time, Notch expression is decreased in prostate and several breast cancers. The surrounding microenvironment determines Notch’s ability to behave as an oncogene or a tumor suppressor gene. Additionally, post-translational changes influence Notch receptors’ intracellular half-lives and ligand affinity [126].

Once the Notch signaling pathway is activated, CSCs are more likely to survive, self-renew, and spread, whereas planned cell death is suppressed. Notch signaling promotes self-renewal as well as metastasis [122,123,124,125,126,127,128].

## 7. Novel Therapeutic Approaches for Targeting CSCs

The targeting of the signaling pathways has recently been proposed as a potential strategy for eradicating CSCs [129,130,131,132]. These techniques are intended to disrupt the signal-transduction pathways related to the self-renewal of CSCs. Blocking these molecular pathways can inhibit the proliferation and cancer progression, target the TME to damage the interconnection between CSCs and cytokines, target the CSC surface markers to determine and seriously affect CSCs, and target metabolism in CSCs [133,134]. All these goals can be accomplished simultaneously. 

However, similar to traditional chemotherapeutics, a significant proportion of the anti-CSC medications that are currently available have several disadvantages that include limited solubility, low stability, high toxicity, and a lack of tissue selectivity. These drawbacks limit the practical applications of such medications. Because CSCs and normal stem cells share many characteristics, typical anti-CSC medicines cannot differentiate between the two types of cells [135,136,137]. 

### 7.1. Targeting CSC Surface Markers

Surface biomarkers are essential for separating, characterizing, and delivering treatment modalities to CSCs. CD44, CD133, and EpCAM have frequently utilized surface biomarkers for CSCs. CD44 is a transmembrane protein found in various CSCS; it performs a critical function in controlling the self-renewal, tumor initiation, treatment resistance, and metastatic features of CSCs [138,139]. The specific upregulation of CD44 in CSCs suggests that CD44 may be a target for CSCs therapeutics. P245, an anti-CD44 antibody, has been shown in murine models to decrease tumor progression and eradicate CSCs [140]. P245 therapy reduced tumor relapse following chemotherapy with doxorubicin (DOX) and cyclophosphamide in human breast cancer mouse models [140].

CD133 is a five-transmembrane glycoprotein that is upregulated in a variety of cancers. CD133, one of the multiple surface biomarkers of CSCs, is required for CSC growth and maintenance, and antibodies targeting CD133 can inhibit CSC progress [141].

The fusion protein dCD133KDEL was shown to be a novel molecular monitoring technique for evaluating the clinical importance of CD133+ cell eradication [142].

EpCAM is another biological target of CSCs; its upregulation might promote tumor progression, metastasis, and treatment resistance [143]. Kubo et al. [143] discovered that when catumaxomab, an EpCAM antibody, was coupled with activated T cells, it was possible to eradicate EpCAM-positive TNBCs and conquer their in vitro chemoresistance.

### 7.2. Inducing CSCs’ Differentiation

CSCs are the focus of differentiation therapy, which alters their stemness to reduce chemotherapeutics resistance [144]. Doxorubicin (DOX), a clinically common chemotherapeutic treatment, and all-trans retinoic acid (ATRA), a potent differentiation agent of CSCs, are concurrently encapsulated in the same nanoparticles using a single emulsion technique; it has been shown that simultaneous delivery-based therapy using ATRA and DOX may effectively transport the medications to both non-CSCs and CSCs to differentiate and eradicate the cancer cells. Differentiating CSCs into non-CSCs can decrease their ability to self-renew and make them more sensitive to chemotherapy; when these two treatments were combined, a significantly improved anticancer effect was observed [145]. Non-CSCs are more sensitive to conventional therapy when their CD44 expression is reduced by lentivirus particles, which was demonstrated by Pham and his colleagues [74].

### 7.3. Targeting Metabolism in CSCs

CSCs have unique metabolic features, including glucose and mevalonate metabolism [146]. For instance, CSCs overexpress hexokinase 2 (HK2), a critical kinase essential for glucose metabolism [147]. Therefore, blocking HK2 may be a strategy for eradicating CSCs. Metformin (MET) boosts chemotherapeutic efficacy in various forms of cancer by inhibiting HK2 [148]. Blocking the mevalonate metabolic pathway with HMG-CoA reductase inhibitors limits CSCs’ growth [148]. Additionally, it has been shown that iron metabolism is critical for CSCs; hence, inhibiting iron metabolism might enhance the therapeutic efficacy against various malignancies [149]. These findings highlight how the unique metabolic features of CSCs can be employed as therapeutic targets.

### 7.4. Targeting the TME

To maintain their function, CSCs require a certain microenvironment [52]. Mesenchymal stem cells (MSCs), immunological cells, cancer-associated fibroblasts (CAFs), autocrine signals, the extracellular matrix, the vascular network, oxygen pressure, and dietary patterns contribute to this unique microenvironment. In addition, this microenvironment might aid in CSC development by promoting CSC-like properties in non-CSCs [150]. Expansion of bone-marrow-derived MSCs in tumor tissue has been shown to govern CSCs through cytokine loops, thus hastening cancer progression. Increased expression of stem cell-specific characteristics by chemokine (C-C motif ligand 2) (CCL2) production in tumor cells is induced by 85 CAFs. It was also discovered that CAFs regulate CSCs either via IL-6 or IL-8. It has been shown that tumor-associated macrophages (TAMs) can promote CSC phenotypes in mouse models of cancer by acting on the epidermal growth factor receptor (EGFR)/signal transducer and activator of transcription 3 (STAT3)/sex-determining region [151].

### 7.5. Target Exosomes of CSCs

Exosomes of CSCs are vesicular membrane structures (CSC-Exos) released by tumor cells. CSC-Exos are nanosized vesicles that facilitate communication between tumor cells and the TME. Four steps contribute to creating CSC-Exos: budding, invagination, multivesicular body formation, and secretion [152]. Exosomes from CSCs serve multiple functions in cancer pathogenesis. CSC-Exos serve as signal transporters for EMT development and transmit these signals to tumor cells to induce tumor spread and invasion. [153,154]. Yang et al. [153]. revealed that cancer-derived exosomes govern cancer cell invasion and metastasis by triggering cancer-associated fibroblasts in the TME, which stimulates the Wnt pathway [153]. Studies have proven that exosomes enhance T cells in evading immune surveillance [155]. CSC-Exos are rich in immunosuppressive proteins, including programmed death-ligand 1 (PDL1). PDL-1 is abundantly produced on the surface of cancer cells and binds to its surface receptor to block T-cell activation, enabling tumor cells to escape the host’s immune response [155]. In addition, exosomes produced from cancer cells suppressed T-cell proliferation via TGF-1, thereby interfering with normal immune function and increasing tumor formation [156]. Chemoresistance has become the greatest obstacle to anticancer therapy. Exosomes generated from HER^2+^ breast cancer cells contain the long non-coding RNA (lncRNA) SNHG14, which is capable of inducing apoptosis and trastuzumab resistance by targeting the B-cell lymphoma-2 gene (Bcl-2/BAX) pathway [157].

Recent research has uncovered the probable mechanisms involved in exosome synthesis, release, and uptake; hence, numerous new techniques to disrupt exosome-mediated signaling may be envisioned. Exosome biogenesis could be inhibited by removing ESCRT components such as HRS, STAM1, and TSG101. In addition to ESCRT machinery, several lipids and lipid-metabolizing enzymes are involved in regulating this mechanism in certain cells [157,158]. By reducing nSMase activity with hydrochloride hydrate (GW4869) or RNAi, exosome synthesis and prion packaging could be diminished [159]. Gernapudi et al. [159] demonstrated that targeting exosomes from preadipocytes inhibited preadipocyte-to-CSCs signaling pathways in early-stage breast cancer and that preadipocyte-derived exosomes promoted tumor progression in vivo, which provided a solid foundation for the importance of exosomal signaling in the TME.

### 7.6. Targeting CSCs’ Quiescence

In response to physiological cell cues, cells can exit and re-enter the cell cycle from a reversible G0 phase known as “cell quiescence” [160]. Studies suggest that quiescent cell gene expression is regulated by transcription factors such as FoxOs (Forkhead Box O) and NFIX (a protein member of Nuclear Factor 1) [161,162]. Signaling molecules that control stem cell quiescence include p53; RB (retinoblastoma protein); the Cdk inhibitors p21, p27, and P57; Notch-related pathways; and several miRNAs (micro-RNAs) [160]. Slowly dividing CSCs could be identified in tumors by using their transcriptional signatures, thereby revealing the tumor heterogeneity in the CSCs’ content [163].

The quiescent state of CSCs protects them from antiproliferative drugs, which play a significant role in CSC-related resistance to conventional therapy. This slow-cycling CSCs subgroup has been targeted in three ways, as mentioned previously. “Locked-out” CSCs can be reactivated by compelling them to re-enter the cell cycle. This approach was offered as a potential advantage in the fight against the disease’s spread. “Locked-out” strategies may provide a risk if existing antiproliferative drugs do not effectively destroy all awakened cancer cells.

For this reason, the tumor may become more genetically and epigenetically complex, making it more resistant to treatments [164]. Some authors have come up with new ways to deal with these issues. One of these is the “locked-in” technique, which entails keeping CSCs in the G0 phase pharmacologically throughout a patient’s life to avoid tumor growth, recurrence, and metastasis. CSCs can be eliminated while they are dormant to prevent their reactivation.

**Figure 6:**
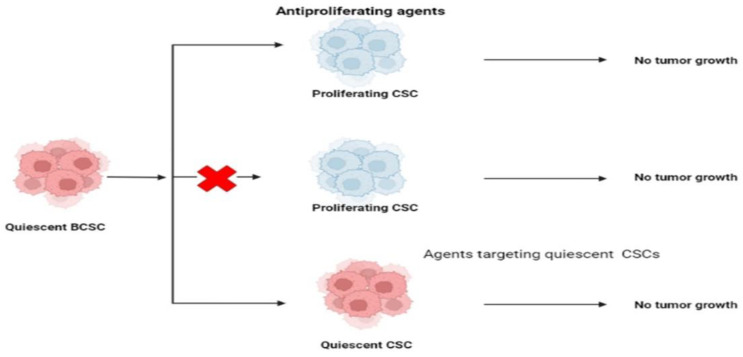
Schematic representation demonstrating that targeting CSCs’ quiescence is a promising approach to tackling drug resistance.

The cyclin-dependent kinase inhibitors p57KIP2, p27KIP1, and p18INK4c might modify the quiescent status of CSCs as well as hematopoietic (HCSs) and chronic myeloid leukemia stem cells. Scientists have demonstrated the importance of these proteins in quiescence and self-renewal activities using knock-in mice models. c-Myc, a transcriptional regulator that regulates the expression of genes associated with cell cycle and proliferative functions, has been connected to the maintenance of quiescence via Fbxw7 (F-box protein), one of the four members of the SCF-type ubiquitin ligase complex. As a result of Fbxw7 ablation increasing imatinib sensitivity in leukemia starting cells, encouraging CSCs to re-enter the cell cycle may boost therapy efficacy [165]. Silencing Fbxw7 in the TNBC cell line MDA-MB-468 resulted in its resistance to paclitaxel treatment, but overexpression of Fbxw7 in the chemotherapeutic drug-resistant cells restored their responsiveness [166]. BCRA1 overexpression has been demonstrated to modify the quiescence of CSCs (Figure 6) (Table 1) [167].

### 7.7. Nanoparticle-Based Drug-Delivery Systems (NDDSs) for Targeting CSCs

**Figure 7:**
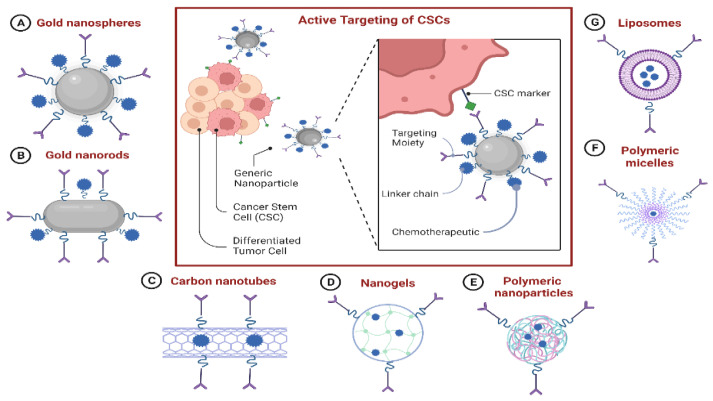
Schematic illustration revealing the various promising types of nanoparticles that contribute to nanoparticle-mediated targeted drug delivery to CSCs.

There is an immediate need to address the issues associated with current anti-CSC medicines, including their low solubility, instability, unfavorable bioavailability, and significant toxicity due to off-target adverse effects. NDDSs may be able to meet this need. Due to their improved permeability and retention properties, NDDSs can passively target malignant cells. Additionally, the CSC-targeting properties of NDDSs could be increased by coating them with appropriate small molecules that bind to overexpressed receptors on the surface of CSCs. A better knowledge of CSC biology and various advancements in nanotechnology have contributed to the development of a growing number of NDDSs for treating multiple types of cancer by eradicating CSCs (Figure 7) (Table 2) [168].

#### 7.7.1. NDDS-Based Delivery of Chemotherapeutics to CSCs

Most CSC-specific chemotherapeutics are presently described as having unsatisfactory in vivo features comparable to conventional chemotherapeutic medicines. Curcumin, a polyphenol derived from the ancient Asian spice turmeric, has been demonstrated to target CSCs by blocking signaling pathways and suppressing the expression of ALDH [169,170]. Nevertheless, its clinical development has been severely limited due to its hydrophobic nature, low in vivo stability, and quick metabolism. Gülçür et al. [170] overcame these limitations by developing a unique nanomicellar formulation of curcumin. Curcumin’s water solubility and stability were significantly increased by enclosing it in sterically stabilized micelles (C-SSM). Additionally, curcumin-encapsulated C-SSM significantly increased curcumin’s efficiency against CSCs.

Type 2 diabetes medication MET has been shown to have an anticancer effect at low dosages through CSC targeting; however, its effectiveness is constrained by its poor bioavailability and non-specific biodistribution. MET-encapsulated trastuzumab-conjugated immunoliposomes (Her-LP-MET) were recently discovered by Lee et al. [171] as a potential method to target CSCs and inhibit their proliferation and spread selectively. HerLP-MET, in conjunction with free DOX, led to a greater tumor recurrence rate than free DOX alone. Sun et al. [144] argued that rationally designed drug-delivery devices could considerably boost CSC eradication by administering conventional chemotherapeutics such as DOX directly into CSCs. They synthesized DOX-tethered gold nanoparticles (DOX-Hyd@AuNPs) and proved that they might decrease tumor progression without expanding the CSC subset in the tumor by enhancing DOX delivery to CSCs. This procedure overcame the CSCs’ inherent resistance to drugs due to P-glycoprotein (P-gp) efflux.

#### 7.7.2. NDDS-Based Delivery of Nucleic Acid Therapeutics to CSCs

In addition to increasing the solubility of low-solubility drugs, NDDSs can strengthen the stability and cellular uptake of cancer-treating molecules such as siRNA, shRNA, and miRNA [165]. AKT2, a key downstream regulator of the phosphatidylinositol 3-kinase (PI3K) pathway, has been associated with the tumorigenesis capacity of CSCs [172]. By suppressing AKT2 with siRNA, tumor formation and relapse can be inhibited. Nonetheless, the quick breakdown of siRNA and its low cellular absorption pose difficulties for siRNA-based therapeutics. Utilizing NDDSs to transport siRNA might be a viable technique for increasing siRNA stabilization and cellular delivery. Recently, novel nanocarrier technology was developed based on Pluronic^®^ F127 micelles and polyethyleneimine (PEI) polyplexes to deliver AKT2 siRNA [173]. This AKT2-siRNA delivery strategy exerted an inhibitory effect on CSC invasion and metastasis.

NF-B is required to maintain CSC characteristics in several types of cancer [174]. As a result, CSCs can be targeted by utilizing RNA interference, such as siRNA and shRNA to downregulate the production of NF-B proteins [175]. Ke et al. [175] designed a carbamate-mannose-modified PEI (CMP) to target the delivery of NF-B shRNA to CSCs; these CMP/NF-B-targeted shRNA nanocomplexes were demonstrated to reduce the percentage of CSCs, limit the formation of mammospheres, suppress cancer invasiveness, and sensitize cancer cells to DOX-loaded micellar nanoparticles. MicroRNAs serve a crucial function in the post-transcriptional control of several cellular processes. MicroRNAs have the capability to control CSCs as well as normal stem cells; however, they also have the ability to dysregulate the mechanism of carcinogenesis [176]. Park et al. [176] created a VP16-Gal4-WPRE integrated systemic amplifier (VISA) delivery system for the miR-34a (TV-miR-34a) plasmid; they revealed that TV-miR-34a was shown to successfully eliminate CSCs and increase treatments’ efficacy against cancerous tissue when paired with docetaxel.

#### 7.7.3. Combinational Delivery of Chemotherapeutics and CSC-Specific Agents

An accumulating body of evidence reveals that tumors are heterogeneous tissues composed of various cell types, including CSCs and non-cancerous stem cells [177]. Although several treatments have been shown to kill CSCs, cancer cells could also naturally transform into CSCs; hence, depleting CSCs alone is not convenient as a therapeutic approach [178]. Rather than focusing on CSCs or non-cancerous stem cells, combination approaches are intended to target both types of stem cells concurrently, which may enhance therapy outcomes.

Kim et al. [177] demonstrated that co-delivery of DOX and SAL in a single constructed cross-linked multilamellar liposomal vesicle (cMLV) strongly inhibited both tumor cells and CSCs, which may have been due to the co-administration of the two drugs to malignant cells via cMLV (DOX + SAL). Likewise, Zhang et al. [178] investigated the therapeutic efficacy of micelles co-loaded with the cytotoxic drug epirubicin (EPI) and the CSC inhibitor staurosporine (STS) in the management of various types of cancer, especially those that had relapsed after traditional chemotherapy. These results indicated that STS/EPI-loaded micelles may be employed to treat naive orthotopic 4T1-luc tumors and their recurrent EPI-resistant counterparts by suppressing tumor cells and the CSC-associated subgroup.

**Table 2:** Recent potential nanoparticle-based drug-delivery systems (NDDSs) for targeting CSCs.

	Drugs	Application/Efficacy	Reference
NDDS-Based Delivery of Chemotherapeutics to CSCs	Curcumin-encapsulated C-SSM. MET-encapsulated trastuzumab-conjugated immunoliposomes (Her-LP-MET). DOX-tethered gold nanoparticles (DOX-Hyd@AuNPs).	Significantly increased curcumin’s efficiency against CSCs [171]. HerLP-MET, in conjunction with free DOX, led to a greater tumor recurrence rate than free DOX alone. Decreased tumor progression without expanding the CSC subset in the tumor by enhancing DOX delivery to CSCs.	[179] [145]
NDDS-Based Delivery of Nucleic Acid Therapeutics to CSCs	Pluronic^®^ F127 micelles and polyethyleneimine (PEI) polyplexes to deliver AKT2 siRNA. Carbamate-mannose-modified PEI (CMP) to target the delivery of NF-B shRNA to CSCs. VP16-Gal4-WPRE integrated systemic amplifier (VISA) delivery system for the miR-34a (TV-miR-34a) plasmid.	This AKT2-siRNA delivery strategy exerted an inhibitory effect on CSC invasion and metastasis. Reduced the percentage of CSCs, limited the formation of mammospheres, suppressed cancer invasiveness, and sensitized cancer cells to DOX-loaded micellar nanoparticles. TV-miR-34a was shown to successfully eliminate CSCs and increase treatments’ efficacy against cancerous tissue when paired with docetaxel [180].	[173] [181] [181] [180]
Combinational Delivery of Chemotherapeutics and CSCs-Specific Agents	Co-delivery of DOX and SAL in a single constructed cross-linked multilamellar liposomal vesicle (cMLV): cMLV (DOX + SAL). Micelles co-loaded with the cytotoxic drug epirubicin (EPI) and the CSC inhibitor staurosporine (STS).	Strongly inhibited both tumor cells and CSCs, which may have been due to the co-administration of the two drugs. STS/EPI-loaded micelles may be employed to treat naive orthotopic 4T1-luc tumors and their recurrent EPI-resistant counterparts by suppressing tumor cells and the CSC-associated subgroup.	[178] [182]

#### 7.7.4. The Benefits and Drawbacks of Using Existing NDDSs against BCSCs

The existing knowledge indicates that NDDSs are viable therapy options that could overcome the drawbacks of conventional therapeutics against CSCs and encourage the growth of prospective anti-CSC medicines. Compared to traditional medication, NDDSs may offer several benefits in the fight against CSCs. For instance, NDDSs can encounter several disadvantages to traditional treatments when used against CSCs [183]. Additionally, while normal stem cells and CSCs might share some traits, including self-renewal, NDDSs may mitigate the cytotoxicity in normal stem cells by exclusively retaining them in tumor tissue via the permeability and retention effect minimizing any influence on normal stem cells [184]. Additionally, the capability of NDDSs to target CSCs might be boosted even more by modifying them with CSC surface-marker-specific ligands/antibodies, which could enhance the anti-CSC activity while decreasing the cytotoxicity of healthy tissues [184].

Additionally, NDDSs have the potential to encapsulate agents that target various cancerous cells, agents that target less-abundant CSCs, and agents that target the TME by a single nanoparticle, allowing such drugs to target the tumor tissue as a single agent; this can alleviate possible issues associated with these medications because their biological properties differ in vivo, thereby preventing them from delivering the intended synergistic activity [185]. Nonetheless, the research on and development of NDDSs to be used against CSCs are immature, and numerous challenges remain. Additional studies on the biological properties of CSCs and the development of increasingly effective NDDSs are required to tackle the drawbacks discovered throughout NDDS applications. To eradicate CSCs from cancer tissue, the targeted NDDSs must reach the CSC-containing sites. Indeed, some CSC subpopulations are found in weakly vascularized regions that are particularly hard to reach with NDDSs [183]. A further disadvantage of NDDSs is that despite numerous proposals to minimize NDDSs’ reticuloendothelial system (RES) uptake, their retention in bypassed organs and cellular uptake by RES macrophages remain significant areas of concern [186].

## 8. Conclusions

Herein, we concluded that there are multiple obstacles to eradicating CSCs, preventing frequent conventional therapeutic resistance and cancer recurrence. Current cancer treatments cannot eradicate progressing tumors because they cannot effectively target CSCs. Although chemotherapy resistance is a critical part of CSC eradication, none of the systematic research conducted to date has been able to apply an effective approach to eliminating them. The lack of adequate investigational strategies has seriously affected the accurate understanding of cancer biology and the apparent function of CSCs in tumor heterogeneity. The current knowledge of scientists regarding CSCs is insufficient to generate an irreplaceable therapeutic approach. However, there is a great deal of hope that future research will reveal curative cancer prevention and treatment methods. Extensive investigation of CSCs’ biology is highly recommended to intensify our knowledge, which consequently would develop adequate therapeutics with the capacity to specifically target CSCs, eliminate them, and prevent cancer relapses and resistance to conventional therapeutics.

## Figures and Tables

**Table 1:** Representation of all sections with article outcomes.

	Origin	Characteristics	Isolation Techniques	Signaling Pathways Governing CSC Behavior	Novel Therapeutic Approaches for Targeting CSCs
Cancer Stem Cells	Cell fusion Horizontal gene transfer Dedifferentiation in cancer cells Tumor Microenvironment (TME)	Autophagy Self-renewal, differentiation, and tumor recurrence Induction of angiogenesis CSCs promote metastasis Radiation and chemoresistance	Isolation with surface markers Side population assay Label-retaining methods Tumorigenicity Aldehyde dehydrogenase assay Spheroid formation assay Stemness gene expression and transcriptional factors	Wnt signaling pathway in CSCs Hedgehog (Hh) signaling Notch signaling	Targeting CSC surface markers Inducing CSC differentiation Targeting metabolism in CSCs Targeting the tumor microenvironment Target exosomes of CSCs Targeting CSCs’ quiescence Nanoparticle-based drug delivery systems (NDDSs) for targeting CSCs

## Data Availability

The datasets used and/or analyzed during the current study are available from the corresponding author upon reasonable request.

## Abbreviations

ABC, ATP-binding cassette; AML, acute myeloid leukemia; ATRA, all-trans retinoic acid; BAA–, BodipyTM-aminoacetate; bFGF, basic fibroblast growth factor; BLBC, basal-like breast cancer; CCL2, C-C motif ligand 2; cMLV, cross-linked multilamellar liposomal vesicle; CNS, central nervous system; CSCs, cancer stem cells; 3D, three-dimensional; Dll, Delta-like; DOX, doxorubicin; EBV, Epstein–Barr virus; EGF, epidermal fibroblast growth factor; EGFR, epidermal growth factor receptor; ELDA, extreme limiting dilution analysis; EPCs, endothelial progenitor cells; ER, estrogen receptor; GSCs, glioma stem-like cells; HGT, horizontal gene transfer; HK2, hexokinase 2; JNK, c-Jun N-terminal kinase; LDA, limited dilution assay; MACS, magnetic cell sorting; MET, metformin; MSCs, mesenchymal stem cells; NO, nitric oxide; PDL1, programmed death-ligand 1; P-gp, P-glycoprotein; PI3K, phosphatidylinositol 3-kinase; PEI, polyethyleneimine; SASP, senescence-associated secretory phenotype; SD-MSCs, mesenchymal stem cells devoid of serum; SP, side population assay; STAT3, signal transducer and activator of transcription 3; STS, staurosporine; TICs, tumor-initiating cells; TIS, therapy-induced senescence; TME, tumor microenvironment; TAMs, tumor-associated macrophages; VISA, VP16-Gal4-WPRE integrated systemic amplifier.
